# Eel's Head Powder Reduces Mild-Moderate Depression in Geriatric Individual: Result from Randomized Controlled Trial Study

**DOI:** 10.1155/2020/4658514

**Published:** 2020-01-07

**Authors:** Karina Shasri Anastasya, Shelly Iskandar, Nur Atik

**Affiliations:** ^1^Graduate School of Biomedical Sciences Master Program, Faculty of Medicine, Universitas Padjadjaran, Bandung, Indonesia; ^2^Department of Psychiatry, Faculty of Medicine, Universitas Padjadjaran/Hasan Sadikin Hospital, Bandung, Indonesia; ^3^Department of Biomedical Sciences, Faculty of Medicine, Universitas Padjadjaran, Bandung, Indonesia

## Abstract

Depression is one of the major problems, and the prevalence is higher among the elderly. The underlying mechanism of depression among this set of the population is multifactorial, and one of the most important factors in the pathophysiology of depression is the level of brain serotonin. Protein deficiency is linked to tryptophan deficiency that is known to be the essential material for the synthesis of serotonin. This randomized controlled trial looked for the effectiveness of eel's head powder administration on plasma tryptophan level and geriatric depression scale (GDS) scores among the elderly population who suffered from depression. The subjects were divided into three intervention groups, including groups that were given 2-week course of 5 mg/kg BW eel's head powder, 10 mg/kg BW eel's head powder, and placebo, respectively. There was a significant difference in plasma tryptophan level and geriatric depression scale between the 10 mg/kg BW group and 5 mg/kg BW group. There was also a significant difference between those given 10 mg/kg BW eel's head powder and those receiving placebo; however, no such difference was found between those in the 5 mg/kg BW eel's head powder group and placebo group. Eel's head powder administration could increase plasma tryptophan level and reduce geriatric depression scale score among older individuals who suffered from depression. Administration of 10 mg/kg BW eel's head powder was significant in increasing plasma tryptophan level and reducing GDS score.

## 1. Introduction

Depression is a common mental disorder worldwide including Indonesia. The prevalence of depression among older individuals is higher compared with other age groups in Southeast Asian country which reaches 17–43%. The prevalence in Indonesia, Vietnam, and Malaysia is 42.5%, 17.2%, and 27.8%, respectively [1, 2].

In the elderly, the most common symptoms of depression are sleeping disturbance, fatigue, lack of interest, hard to concentrate, decreased appetite, and loss of life purpose and expectation [3, 4]. Recently, abnormalities in monoamine neurotransmitters such as norepinephrine (NE), dopamine (DA), serotonin (5-Hydroxytryptamine), and histamine are considered to play significant roles in the pathophysiology of depression [5, 6]. Low serotonin level is thought to be the most influential factor predisposing to the occurrence of depression, especially among this particular population [7]. Several clinical trials showed that consumption of high-protein food, especially tryptophan that can increase brain serotonin, is recommended for depressive patients [8–10]. Alteration in tryptophan metabolism in older individuals, disturbance in nutrient intake, and the existence of chronic comorbidities have led this specific population to a vulnerable state of being affected by depression. Moreover, administration of eel's head powder that carries a high amount of protein, especially tryptophan, might be beneficial, and it is known that tryptophan will subsequently recover serotonin balance [11–16]. Therefore, the aim of this study is to determine the effectiveness of eel's head powder to reduce depression in the frail elderly group through increment of tryptophan level in blood.

## 2. Materials and Methods

All procedures performed in this study were approved by the Research Ethics Committee, Universitas Padjadjaran, Indonesia (80/UN6.KEP/EC/2018).

### 2.1. Eel Powder Production

The fresh eel's head (*Anguilla bicolor bicolor*) was collected from Pelabuhanratu, Sukabumi, West Java, Indonesia. The head of the eel was washed with freshwater, followed by boiling, pressing, drying, grinding, packaging, and packing. We use fresh eel powder for this research.

### 2.2. Human Subject Participants

The study population consisted of all elderly residents at one of nursing home, Bandung, the capital city of West Java Province. Convenience sampling was accomplished by recruiting the subjects with specific inclusion and exclusion criteria. The inclusion criteria were older than 60 years old and had mild and moderate depression and geriatric depression scale (GDS) ≥ 10. Those who suffered from cognitive impairment (Mini Mental State Examination (MMSE) < 24), neurological disorder, diabetes mellitus, severe depression, and treated by antidepressant medications are excluded. The study samples were randomized into three groups: (1) received 5 mg/kg BW eel's head powder, (2) received 10 mg/kg BW eel's head powder, and (3) received a placebo. The intervention was given for 14 days.

At day 0, the patients were randomly assigned to their respective group, their body weight was measured, and 3 cc preinterventional blood sample was obtained, and the patients were interviewed using the GDS and MMSE; at day 1, the patients were given either eel's head powder or placebo accordingly and took it regularly for 14 days. At day 15, 3 cc blood sample was obtained from the blood vessel (intravenous), and the second GDS test was performed. All the subjects were informed about the study procedures, and written consent was obtained from all patients.

### 2.3. Cognitive and Depression Measurement

Screening of cognitive impairment uses MMSE that has high sensitivity and specificity. GDS 15 is used to measure self-rated depressive symptoms. The score can be categorized as without depression (0–4), mild depression (5–8), moderate depression (9–11), and severe depression (12–15). Validity and reliability has been tested in Indonesian language [17].

### 2.4. ELISA Procedure

The blood was sent to an accredited private laboratory in Bandung, Indonesia. Tryptophan was analysed using tryptophan ELISA kit (LDN) BA E-2700 for each participant.

### 2.5. Statistical Analysis

Data are expressed as mean ± standard deviation (SD) in case of normally distributed. Plasma values of tryptophan and GDS score from all groups were compared using the ANOVA test and followed by Student's *t*-test, with a significance level of 0.05. The comparison of pre- and posttryptophan plasma levels and GDS in the placebo, 10 mg/kg BW, and 5 mg/kg BW eel treatment groups were obtained by testing the data using paired *T* tests when the data are normally distributed (tryptophan plasma) and alternative Wilcoxon test when the data are not normally distributed (GDS). We performed Pearson's correlation test to analyse correlation between postintervention plasma tryptophan level and GDS. The data were processed using SPSS version 24.0 for Windows [18].

## 3. Results and Discussion

### 3.1. Results

The total participants who were recruited in this study were 42 participants. Three subjects from the placebo group and 3 subjects from the 10 mg/kg BW eel's head powder group were dropped out. They refused to consume eel's head powder capsule due to unpleasant smell of the eel's head powder. Thirty-six subjects were successfully included: eleven subjects received 10 mg/kg BW eel's head powder, fourteen subjects received 5 mg/kg BW eel's head powder, and eleven subjects received the placebo.

#### 3.1.1. Results of the Normality Test

Analysing the normality of our data using Saphiro–Wilk showed that most of the data were normally distributed. The data were nonnormally distributed in the pre- and posttest GDS (Table 1).

#### 3.1.2. The Difference of Plasma Tryptophan Level and Geriatric Depression Scale among the Study Groups

We compared the results of tryptophan plasma and GDS before and after intervention with the 10 mg/kg BW eel's head powder group, 5 mg/kg BW eel's head powder group, and placebo. Between those groups, there were statistically significance differences (*p* < 0.05) in the postintervention plasma tryptophan level and GDS parameters. Group receiving 10 mg/kg BW eel's head treatment yielded higher postintervention plasma tryptophan level and lower GDS score than the other groups (Table 2).

Further, analysis of the pre- and posttest tryptophan plasma levels and GDS in the 10 mg/kg BW eel's head powder showed significantly different, whereas the tryptophan plasma was increased, and the GDS was decreased after treatment (Table 3). However, we could not find any difference in the 5 mg/kg BW eel's head powder and placebo group (Tables 4 and 5).

**Figure 1:**
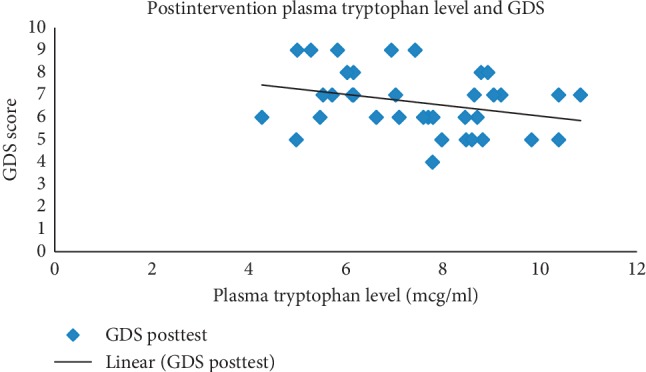
Correlation between postintervention plasma tryptophan level and GDS.

Finally, we analysed the tryptophan plasma level and GDS score after treatment with the 10 mg/kg BW to see any correlation among them. We found that there was a negative correlation between the tryptophan plasma level and the GDS score in the subjects (Figure 1 and Table 6).

### 3.2. Discussion

Our present study showed administration of 10 mg/kg BW eel's head powder was significant in increasing plasma tryptophan level and reducing depression symptoms in the elderly subjects. Additionally, we showed a significant negative correlation among these two parameters which supports the previous finding.

The findings can be explained by correlation between age and concentrations of immune markers and neuropsychiatric symptoms in elderly persons. Increased inflammation in the aging process was related with lower tryptophan concentrations and increased kynurenine level [11]. According to the monoamine hypothesis of depression, this depletion of tryptophan could lead to the insufficient synthesis of neurotransmitter to a depressive mood state [19]. Meanwhile, another related study stated that acute tryptophan depletion might induce relapse of depression in patients whose serotonergic mechanism may already be compromised [14].

Eel's head was found to contain the highest amount of protein, that is rich in tryptophan, among its other body parts. It is also essential to consider that the supply of tryptophan to the brain depends on the amount of free tryptophan in the blood and other amino acids which compete to cross the blood-brain barrier. Besides the capsules of eel's head powder do contain tryptophan, it also contains several substances, including EPA, DHA, and other amino acids [3]. Since the supplementation of tryptophan is not an absolute factor that affects the patient's mood, we also have to take into account other neurotransmitters that influence someone's mood, namely, dopamine and epinephrine [5].

Dopamine is synthesized from tyrosine amino acid at the presynapses terminal and released to the synapse space subsequently. Tyrosine is oxidized into dopa, underwent further decarboxylation into dopamine, and oxidized into norepinephrine. Norepinephrine is methylated into epinephrine. The final products of this biosynthesis are epinephrine and norepinephrine or as a group is called catecholamine that can be converted from dopamine in particular tissues [4].

Furthermore, EPA and DHA omega fatty acids in eel's head powder influence serotonin status by enhancing its production and reception; and deficiency of DHA is linked with dysfunction and impaired transmission of serotonin, norepinephrine, and dopamine. Thus, omega fatty acids might also improve GDS score by enhancing production and reception of related neurotransmitter together with tryptophan as a sole precursor of serotonin, as well as tyrosine in the production of dopamine [5, 8].

Therefore, administration of an adequate amount of tryptophan supplied from the eel's head powder in the elderly patient could be beneficial to compensate for the reduced tryptophan level and fulfil the daily recommendation of tryptophan intake as much as 4 mg/kg BW, as recommended by WHO. EPA, DHA, and other amino acids in eel's head powder also increase its benefit in reducing depression symptoms [5, 19, 20].

## 4. Conclusion

Administration of eel's head powder can increase plasma tryptophan level and reduce geriatric depression scale score among elderly with depressive disorder. Daily administration of 10 mg/kg BW eel's head powder has more significant effects in increasing plasma tryptophan level and reducing geriatric depression scale score as compared with 5 mg/kg BW/day and placebo.

## Figures and Tables

**Table 1:** Normality test result.

Variables	Group	*p* value	Data distribution
Pretest plasma tryptophan level	Overall	0.067	Normal
Posttest plasma tryptophan level	Overall	0.495	Normal
Pretest GDS	Overall	0.004^*∗∗*^	Nonnormal
Posttest GDS	Overall	0.017^*∗∗*^	Nonnormal
Pretest plasma tryptophan level	10 mg	0.492	Normal
Posttest plasma tryptophan level	10 mg	0.871	Normal
Pretest GDS	10 mg	0.158	Normal
Posttest GDS	10 mg	0.009^*∗∗*^	Nonnormal
Pretest plasma tryptophan level	5 mg	0.174	Normal
Posttest plasma tryptophan level	5 mg	0.795	Normal
Pretest GDS	5 mg	0.205	Normal
Posttest GDS	5 mg	0.107	Normal
Pretest plasma tryptophan level	Placebo	0.132	Normal
Posttest plasma tryptophan level	Placebo	0.213	Normal
Pretest GDS	Placebo	0.042^*∗∗*^	Nonnormal
Posttest GDS	Placebo	0.108	Normal

*p* value was calculated based on Saphiro–Wilk test; the *p* value of >0.05 reflects normal data distribution, while the *p* value of <0.05 is considered as nonnormally distributed data.

**Table 2:** Comparison between pre- and postintervention plasma tryptophan level and GDS between the 10 mg/kg BW eel's head powder group, 5 mg/kg BW eel's head powder group, and placebo group.

Variables	Groups			*p* value
	10 mg/kg	5 mg/kg BW	Placebo	
	*N* = 11 (in mcg/ml)	*N* = 14 (in mcg/ml)	*N* = 11 (in mcg/ml)	
*Pretest plasma tryptophan level*				
Mean ± SD	6.80 ± 1.78	6.75 ± 2.43	7.79 ± 2.38	**0.466**
Range (min–max)	4.30–10.81	3.63–10.75	4.41–13.67	

*Posttest plasma tryptophan level*				
Mean ± SD	8.66 ± 0.93	7.23 ± 1.95	6.64 ± 1.37	**0.012** ^*∗∗*^
Range (min–max)	7.10–10.39	4.27–10.84	4.98–8.93	

*Pretest GDS*				
Mean ± SD	6.90 ± 1.375	7.00 ± 1.519	7.00 ± 1.897	**0.983**
Range (min-max)	5.00–9.00	5.00–10.00	5.00–10.00	

*Posttest GDS*				
Mean ± SD	5.72 ± 0.78	6.85 ± 1.29	7.36 ± 1.50	**0.012** ^*∗∗*^
Range (min–max)	5.00–7.00	4.00–9.00	5.00–9.00	

For continuous variables, data were analysed using one-way ANOVA test if the distribution was normal and had homogenous variants, and Kruskal–Wallis test was used for nonnormally distributed data. The *p* value of <0.05 was considered statistically significant. ^*∗∗*^Statistically significant *p* value of <0.05.

**Table 3:** Comparison between pre- and postintervention plasma tryptophan level and GDS score among intervention in patients receiving 10 mg/kg BW eel's head powder.

Variables	Groups		*p* value
	Pretest	Posttest	
	*N* = 11 (in mcg/ml)	*N* = 11 (in mcg/ml)	
*Plasma tryptophan*			
Mean ± SD	6.80 ± 1.78	8.66 ± 0.94	**0.007** ^*∗∗*^
Range (min–max)	4.30–10.81	7.10–10.39	

*GDS*			
Mean ± SD	6.90 ± 1.38	5.72 ± 0.79	**0.027** ^*∗∗*^
Range (min–max)	5.00–9.00	5.00–7.00	

For continuous data, the *p* value was obtained from dependent Student's *t*-test if the data distribution was normal, and alternatively Wilcoxon test was used for nonnormally distributed data. *p* value of <0.05 was considered statistically significant. ^*∗∗*^Statistically significant *p* value of <0.05.

**Table 4:** Comparison between pre- and postintervention plasma tryptophan level and GDS score among intervention in patients receiving 5 mg/kg BW eel's head powder.

Variables	Groups		*p* value
	Pretest	Posttest	
	*N* = 14 (in mcg/ml)	*N* = 14 (in mcg/ml)	
*Plasma tryptophan*			
Mean ± SD	6.75 ± 2.44	7.23 ± 1.96	**0.245**
Range (min-max)	3.63–10.75	4.27–10.84	

*GDS*			
Mean ± SD	7.00 ± 1.519 mcg/ml	6.85 ± 1.292 mcg/ml	**0.635**
Range (min-max)	5.00–10.00	4.00–9.00	

For continuous data, the *p* value was obtained from dependent Student's *t-*test if the data distribution was normal, and alternatively Wilcoxon test was used for nonnormally distributed data. *p* value of <0.05 was considered statistically significant.

**Table 5:** Comparison between pre- and postintervention plasma tryptophan level and GDS score among intervention in patients receiving placebo.

Variables	Groups		*p* value
	Pretest	Posttest	
	*N* = 11 (in mcg/ml)	*N* = 11 (in mcg/ml)	
*Plasma tryptophan*			
Mean ± SD	7.79 ± 2.39	6.64 ± 1.37	**0.086**
Range (min-max)	4.41–13.67	4.98–8.93	

*GDS*			
Mean ± SD	7.00 ± 1.90	7.36 ± 1.50	**0.214**
Range (min-max)	5.00–10.00	5.00–9.00	

For continuous data, the *p* value was obtained from dependent Student's *t-*test if the data distribution was normal, and alternatively Wilcoxon test was used for nonnormally distributed data. *p* value of <0.05 was considered statistically significant.

**Table 6:** Table of correlation analysis of postintervention tryptophan plasma level and GDS.

Variables	Correlation	*R*	*p* value
Correlation of postintervention plasma tryptophan level	*Spearman*	−**0.300**	**0.038** ^*∗∗*^

*p* value of <0.05 was considered statistically significant. ^*∗∗*^Statistically significant *p* value of <0.05. *R*: correlation coefficient.

## Data Availability

The data used to support the findings of this study are available from the corresponding author upon request.
