# Corrigendum to “Eel's Head Powder Reduces Mild-Moderate Depression in Geriatric Individual: Result from Randomized Controlled Trial Study”

**DOI:** 10.1155/2021/4127204

**Published:** 2021-01-04

**Authors:** Karina Shasri Anastasya, Shelly Iskandar, Dewi Marhaeni Diah Herawati, Nur Atik

**Affiliations:** ^1^Graduate School of Biomedical Sciences Master Program, Faculty of Medicine, Universitas Padjadjaran, Bandung, Indonesia; ^2^Department of Psychiatry, Faculty of Medicine, Universitas Padjadjaran/Hasan Sadikin Hospital, Bandung, Indonesia; ^3^Department of Public Health, Faculty of Medicine, Universitas Padjadjaran, Bandung, Indonesia; ^4^Department of Biomedical Sciences, Faculty of Medicine, Universitas Padjadjaran, Bandung, Indonesia

In the article titled “Eel's Head Powder Reduces Mild-Moderate Depression in Geriatric Individual: Result from Randomized Controlled Trial Study” [1], there was an error in the Materials and Methods section. In Section 2.1, Eel Powder Production, “Pelabuhanratu, Sukabumi, West Java, Indonesia,” should be corrected to “Banyuwangi, East Java, Indonesia.” Dr. Dewi Marhaeni was previously acknowledged in the acknowledgements statement. However, due to author miscommunication, she should have been listed as an author. The corrected author list is shown above. The authors apologize for these mistakes.
